# Molecular Dynamics Study of Self-Assembly of Aqueous Solutions of Poly[9,9-bis(4-Sulfonylbutoxyphenylphenyl) Fluorene-2,7-diyl-2,2’-Bithiophene] (PBS-PF2T) in the Presence of Pentaethylene Glycol Monododecyl Ether (C_12_E_5_)

**DOI:** 10.3390/ma9050379

**Published:** 2016-05-18

**Authors:** Beverly Stewart, Hugh Douglas Burrows

**Affiliations:** Centro de Química de Coimbra, Chemistry Department, University of Coimbra, Coimbra 3004-535, Portugal; burrows@ci.uc.pt

**Keywords:** molecular dynamics simulation, conjugated polyelectrolytes, nonionic surfactant, phase formation, temperature dependence

## Abstract

Results are presented using molecular dynamics (MD) of the self-assembly of the conjugated polyelectrolyte poly[9,9-bis(4-sulfonylbutoxyphenylphenyl) fluorene-2,7-diyl-2,2’-bithiophene] (PBS-PF2T) with 680 mM pentaethylene glycol monododecyl ether (C_12_E_5_) in water. Simulations are used to examine the interaction between PBS-PF2T and C_12_E_5_ and suggest a break-up of PBS-PF2T aggregates in solution. These systems are dominated by the formation of cylindrical phases at temperatures between 0 °C and 20 °C and also between 45 °C and 90 °C. More diffuse phases are seen to occur between 20 °C and 45 °C and also above 90 °C. Simulations are related to previous computational and experimental studies on PBS-PF2T aggregation in the presence of tetraethylene glycol monododecyl ether (C_12_E_4_) in bulk and thin films.

## 1. Introduction

Water soluble conjugated polyelectrolytes (CPEs) [1,2] are a versatile class of materials, for which solubility is often due to the presence of hydrophilic side chains which incorporate, for example, non-charged oligoethyleneoxide [3,4] or charged alkylammonium groups [5,6]. The interactions observed between CPEs and water form the basis of this work. Conjugated polyelectrolytes have applications in charge transport and blocking layers [7,8] chemosensors [9,10] and biosensors [11,12]. Water-polymer aggregation is also a prerequisite for conjugated polyelectrolyte deposition for applications such as layer by layer assemblies [13,14] and Langmuir-Blodgett films [15,16].

CPEs are, however, generally subject to issues surrounding their solubility, are difficult to dissolve down to a single molecule level and have a propensity for aggregation without precipitating [17]. Several approaches have been implemented to resolve this issue and, therefore, recover the favorable optical and electronic properties of CPEs. It was first shown that the presence of bulky counterions can disrupt aggregation in charged polythiophenes where there is a change from a red aggregate suspension to a yellow solution [18,19]. This was also applied to sugar substituted poly(*p*-phenylene ethynylene) by Lavigne *et al*. [20], where it was shown that color changes could be induced by a wide range of ionic and non-ionic surfactants. It was these observations that led to such phenomena being termed “*surfactochromism*”. It has also been shown that fluorescence enhancement occurs when a negatively charged poly(*p*-phenylene vinylene) polymer forms a complex with a cationic surfactant in water [21]. This strategy was expanded by Burrows *et al*. [22] by demonstrating that the fluorescence of anionic polyfluorene is enhanced in the presence of a nonionic surfactant, pentaethylene glycol monododecyl ether (C_12_E_5_). The basis for the strategy of introducing surfactants is centered on the fact that water is observed to quench CPE fluorescence whereas surfactant presence restores it to a level observed in organic solvents by forming an isolating layer between the polymer and surrounding water. It was also found that the electronic transition in fluorene is relatively insensitive to solvent polarity [23] and based on comparisons with spectral properties of other fluorene copolymers in both thin films and as isolated chains in solution [24,25]. It is suggested that emission in the clusters is red-shifted and of lower intensity due to π-π interaction and that spectral changes observed upon addition of surfactant are due to cluster breakup and subsequent decrease of chain interactions. It is also suggested that the reduced fluorescence quantum yields observed in water may be attributed to π-π interactions between aromatic backbones on neighboring CPEs leading to loosely defined CPE clusters [26]. 

**Figure 1:**
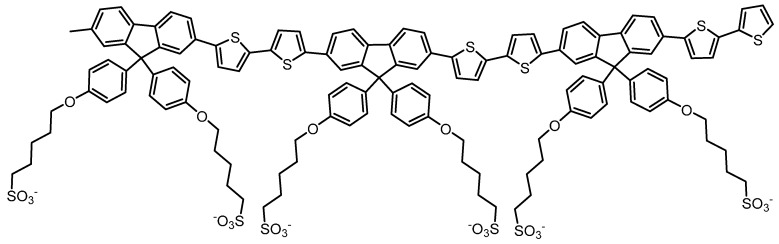
Chemical structure of PBS-PF2T polymer, representative of the trimer used in the MDS simulations.

Using Molecular Dynamic Simulation (MDS) methods we show how the introduction of C_12_E_5_ to a simulation cell containing poly[9,9-bis(4-sulfonylbutoxyphenylphenyl) fluorene-2,7-diyl-2,2’-bithiophene] (PBS-PF2T) (see Figure 1) results not only in the inhibition of PBS-PF2T aggregation but also in the formation of well defined PBS-PF2T:surfactant assemblies at certain temperatures. These simulations are an expansion on previous studies of the interaction of PBS-PF2T and tetraethylene glycol monodecyl ether (C_12_E_4_) by Knaapila *et al*. [27]. These studies included examination of PBS-PF2T mixed into aqueous C_12_E_4_ in bulk and thin films. Blue shifts observed in fluorescence measurements demonstrated a breakup of PBS-PF2T aggregates in bulk aqueous C_12_E_4_. Small angle X-ray scattering (SAXS) was employed and it was revealed that this mixture shows a similar phase behavior to that which has been observed for binary mixtures of pure surfactant with water, comprising a micellar phase below approximately 20 °C, a lamellar liquid crystalline phase between 20 °C and 70 °C and a suggested coexisting water and liquid surfactant solution above 70 °C. In wet films of PBS-PF2T in C_12_E_4_, grazing incidence small angle X-ray scattering (GISAXS) showed that the phase window for lamellar phase formation is rather narrower at around 30–34 °C and weakly ordered phases exist above and below these temperatures. These phases were considered to be isotropic. However, lamellae become aligned in a stacked manner on the surface whether approached from high or low temperatures. Dry films were found to be disordered, but, by maintaining a temperature of 30–34 °C and switching relative outside humidity between 32% and 100%, could be reversibly ordered and disordered.

Studies presented here are based on the observation that at higher surfactant concentrations binary water-surfactant systems can show a range of lyotropic liquid crystalline (LC) phases [28]. This is an important phenomenon interrelated to the potential application of LC π-conjugated polymers for enhanced charge transport in plastic electronic devices and this has already been demonstrated [29]. Effects on surfactant cloud points have been attributed to the incorporation of anionic CPEs within mesophases formed in the binary nonionic alkyloxyethylene surfactant-water systems [30]. Therefore, here we demonstrate a continuation of the study concerning PBS-PF2T in C_12_E_4_-water systems and examine the effect of C_12_E_5_ as the surfactant. MDS provide the basis for all investigations.

PBS-PF2T was selected for both this investigation and the previous study involving C_12_E_4_ as an archetypal CPE [27]. Fluorene-bithiophene alternating copolymers, for example poly(9,9-dioctylfluorene-*co*-bithiophene) are commonly used in molecular electronic devices as charge transport layers [31] while the charged sulfonate groups on the termini of each side chain provide solubility in polar solvents. As mentioned previously, aggregation is a known disadvantage of polyelectrolytes. In water, PBS-PF2T forms a clear transparent solution although small angle scattering measurements indicate the presence of ribbon-like aggregates in 0.05%–0.5% surfactant [32]. The polymers were measured as being around 6 nm in length whereas the ribbons were found to be approximately 2.5 nm in width with their lateral dimension extending beyond 30 nm.

The effect of the introduction of C_12_E_5_ to solutions of the anionic conjugated polyelectrolyte poly{1,4-phenylene-[9,9-bis(4-phenoxybutylsulfonate)]fluorine-2,7-diyl} (PBS-PFP) has previously been examined. PBS-PFP forms a metastable dispersion of polymer aggregates upon stirring in water. It was found however, that these aggregates were broken up to give stable solutions upon C_12_E_5_ addition [22,33]. Nonionic surfactants containing polyoxyethylene chains also demonstrate interesting phase behavior in water, which results in complicated binary and ternary phase diagrams [25,34,35,36]. In the case of C_12_E_5_, dilute lamellar and isotropic liquid phases, thought only to exist in complex surfactant systems, can also be observed in simple binary mixtures [32,35]. The phase diagram for C_12_E_5_ with water is similar to that of C_12_E_4_ although with phase boundaries shifted to higher temperatures. Previous studies of temperature effects on C_12_E_4_-PBS-PF2T surfactant-CPE systems will be used comparatively here to describe the effect of changing surfactant on phase formation in addition to the changes of temperature in C_12_E_5_-PBS-PF2T.

Here, we consider a system of PBS-PF2T mixed with the aqueous surfactant. As in previous studies, with these systems we consider a low polymer fraction (12 mM = 0.5% (*v*)) but increase the surfactant concentration up to a level where liquid crystal phases are anticipated to start to form (680 mM = 26% (*v*)). Previous investigations have demonstrated how PBS-PF2T can be incorporated into a standard surfactant at high mole fraction in bulk and thin films such that the polymer experiences similar optical changes to those in low surfactant fraction, while the surfactant follows its characteristic liquid crystalline behavior [27].

## 2. Results

MDS were used to obtain detailed information on the interactions between PBS-PF2T and C_12_E_5_. A simulation cell of specifications described in Table S1 (Supplementary Materials, SM) was used to observe the effect of temperature on a solution of 680 mM C_12_E_5_ containing two equivalents of PBS-PF2T. Simulations were carried out at 0 °C, 10 °C, 20 °C, 45 °C, 70 °C and 90 °C. These simulations are compared to previous simulations in C_12_E_4_ and also with a prior single simulation of 10 equivalents of PBS-PF2T which demonstrated aggregation in the absence of C_12_E_5_.

**Figure 2:**
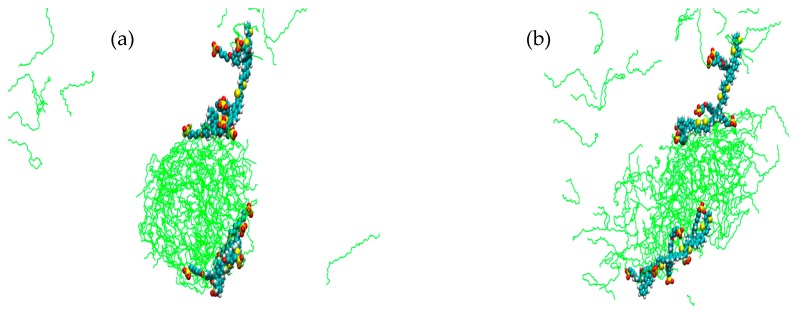
Simulation cell representation (**a**) front view; and (**b**) side view of 680 mM C_12_E_5_ with two equivalents of PBS-PF2T at 0 °C after 10 ns, system **1**. (PBS-PF2T is shown in van der Waals representations and solvent is omitted for clarity).

**Figure 3:**
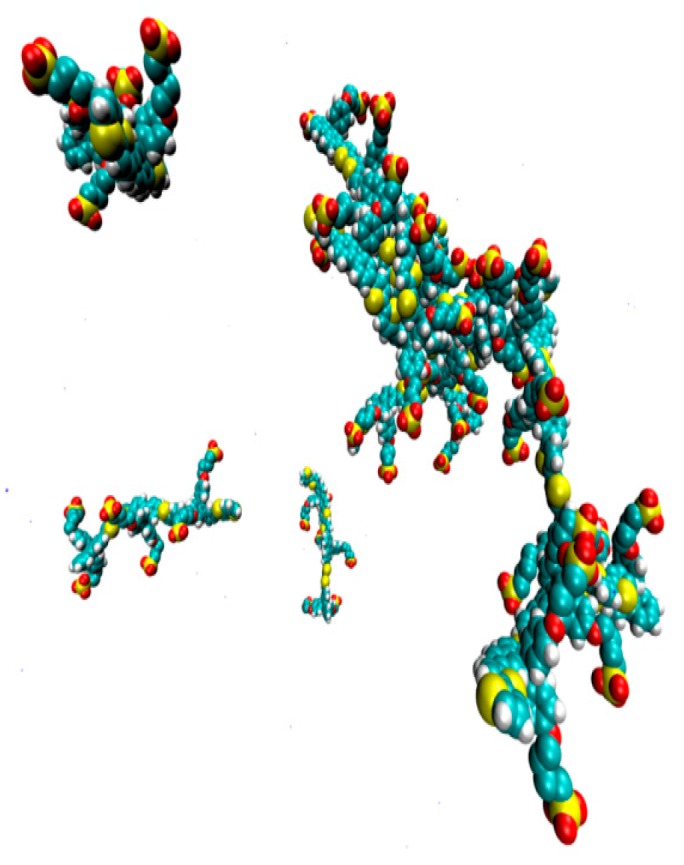
Simulation cell at 20 ns of ten equivalents of PBS-PF2T in water, PBS-PF2T in van der Waals representation [27].

For simulations **1**–**6** each run was carried out from the same starting arrangement of molecules, (considered as structure A). Figure 2 shows simulation cell **1** after a run of 10 ns at 0 °C; an interesting observation here is that, whilst there is a distinctive formation resembling a cylindrical structure, there also seems to be a propensity for one of the PBS-PF2T equivalents to remain partially dissociated in the surrounding solvent. This run was extended to 20 ns (Figure S4a,b) not only to further observe this dissociation but also to determine persistence of this cylindrical phase. The presence of the rod-like PBS-PF2T:C_12_E_5_ assembly, with dimensions of 11.5 × 3.7 nm^2^, suggests that phase formation is very specific, as opposed to a non-defined, diffuse or isotropic phase. For comparison, Figure 3 shows a previously studied simulation of 10 equivalents of PBS-PF2T in water (simulation cell details Supplementary Materials) [27]; where it is clear that, in the absence of any surfactant, PBS-PF2T units interact with each other, resulting in the significant formation of a large PBS-PF2T assembly. It should be noted that this aggregate shows a shape which is similar to that predicted by scattering measurements [27], as the assembly is dominated by a long chain-like structure, (Figure S10) which measures ~95 nm in length. It is this type of cluster formation that, it is suggested, leads to a reduction in the fluorescence quantum yield and red-shifted fluorescence maxima. By comparing Figure 2 and Figure 3, it can be observed that the presence of C_12_E_5_ affords a significant inhibition of aggregation between PBS-PF2T which occurs in a water only environment.

Upon an increase of 10 °C, system **2**, a cylindrical phase is again observed, which is of similar dimensions as was seen at 0 °C; here it is 10.2 × 3.2 nm^2^ in which the two PBS-PF2T equivalents exist at a considerable distance from one another on opposing sides of the rod structure (Figure S1). In this case it can be seen that there is an inhibition of interactions between the PBS-PF2T units and, instead, there is specific assembly formation. This is compared with a similar simulation which was performed with PBS-PF2T in 680 mM C_12_E_4_, (Figure S11a,b) which showed a micellar formation at this temperature. This demonstrates that the type of surfactant present influences the phase behavior of the CPE: Surfactant compositions and consequently the type of phase formed.

**Figure 4:**
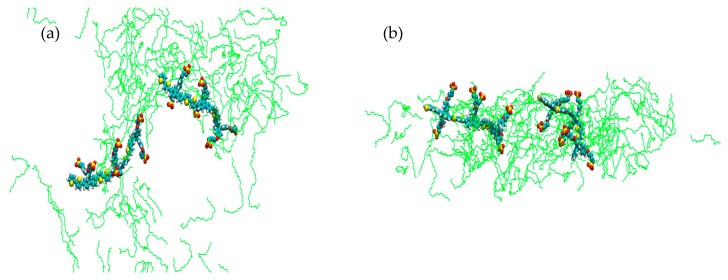
Simulation cell representation of (**a**) front view; and (**b**) side view of 680 mM C_12_E_5_ with two equivalents of PBS-PF2T at 20 °C after 10 ns, system **3**. (PBS-PF2T is shown in van der Waals representations and solvent is omitted for clarity).

After a further increase in temperature to 20 °C a slightly different phase is observed; here a more disperse system is seen, where the surfactant forms a layer ~2 nm deep with what appears to be a circular hydrophilic area which has some resemblance of a reverse micelle. In this simulation it is also clear that the interaction between the PBS-PF2T units is suppressed; Figure 4. At the same temperature, when C_12_E_4_ is present as the surfactant, (Figure S12a,b), a cylindrical assembly can be seen, which is similar to that observed for C_12_E_5_ at 0 °C and 10 °C, this is again indicative of surfactant presence inducing specific phase formation.

At 45 °C the cylindrical structure formation seen at lower temperatures is reformed, system **4** (Figure S2). In this instance, the PBS-PF2T equivalents again remain at a distance from each other and are embedded within the cylindrical structure. However, it can also be observed that the remaining surfactant molecules also form rod-like structures, (Figure S7e,f). The assembly which contains the two PBS-PF2T units is of dimensions 11.6 × 3.54 nm^2^ so is of a similar size to the other rod-like structures observed thus far. It is very possible that at higher surfactant concentrations these cylinder-like structures containing C_12_E_5_ become denser and well defined resulting in the formation of multiple rod-like PBS-PF2T:C_12_E_5_ assemblies. At the same temperature, the simulation of PBS-PF2T in C_12_E_4_ showed a very similar phase formation, (Figure S13a,b).

At 70 °C, system **5**, the distinctive cylinder-like structure persists, this time of dimension 13.2 × 3.7 nm^2^, in which the two PBS-PF2T species are maintained at a distance from each other and do not interact (Figure S3). This formation is similar to that observed in the previous case where C_12_E_4_ is present as the surfactant. However, in the case of C_12_E_4_, dissociation of one of the PBS-PF2T units is observed (Figure S14a,b). It is possible that at this temperature the nature of the surfactant present in the aqueous environment may influence the solubility of the polyelectrolyte and this is an important observation as it is not only the inhibition of cluster formation which promotes the use of conjugated polyelectrolytes in applications mentioned herein but there is also the issue of solubility as a whole. If surfactant type can influence solubility to such an extent as to have singular chains within solution and not part of an assembly, there is the potential for even greater control over the system and hence the applicability of these species to a wider range of technologies.

**Figure 5:**
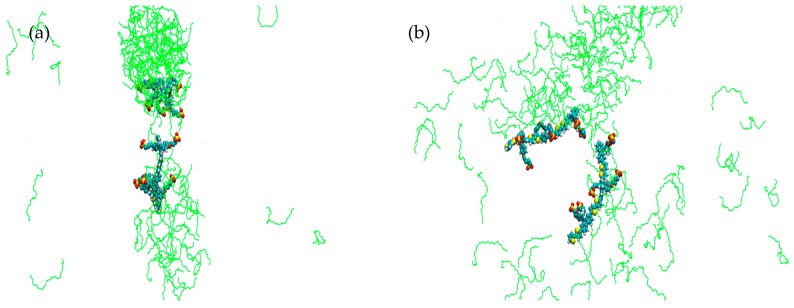
Simulation cell representation of (**a**) front view; and (**b**) side view of 680 mM C_12_E_5_ with two equivalents of PBS-PF2T at 90 °C after 10 ns, system **6**. (PBS-PF2T is shown in van der Waals representations and solvent is omitted for clarity).

Upon a final increase in temperature to 90 °C, system **6**, the overall phase formation becomes somewhat more diffuse and seems in appearance to lie somewhere between an isotropic and the defined cylindrical formations seen so far, Figure 5. The appearance of the loosely defined rod-like formation does bare some resemblance to that seen for a simulation carried out at the same temperature where C_12_E_4_ was the surfactant although in the case of C_12_E_4_ the phase formation is much more distinctive (Figure S15a,b). This simulation also shows some significant dissociation of one of the PBS-PF2T, as this occurs in both C_12_E_5_ and C_12_E_4_ environments there is a strong suggestion of a temperature dependence on solubility.

**Figure 6:**
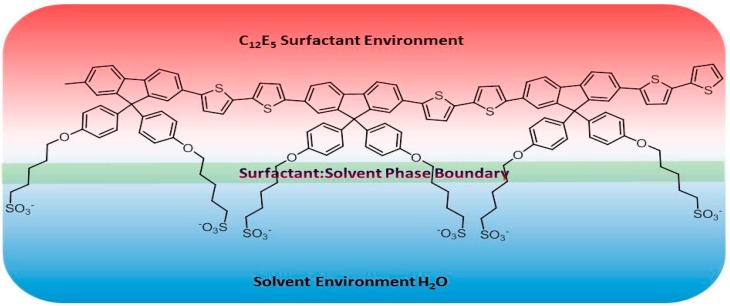
A schematic representation of the specific interactions occurring within the PBS-PF2T assemblies. The backbone interacts predominantly with the surfactant whereas the side chains interact more strongly with the surrounding solvent.

**Figure 7:**
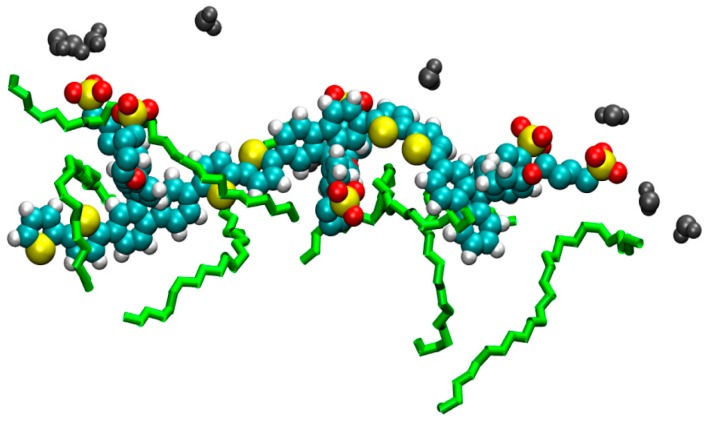
The polymeric part of PBS-PF2T surrounded by C_12_E_5_ (green) and sulfonate groups interacting with water (grey van der Waals).

It is also apparent in each of the simulations—also observed in the previous studies concerning C_12_E_4_ [27]—that there is a specific orientation of the PBS-PF2T units. The polymer is principally incorporated into the surfactant. This incorporation is, however, rather particular. The charged side chains, explicitly the charged sulfonate termini, of the polymer maintain close contact with the surrounding solvent whereas the backbone remains strongly embedded into the surfactant environment. These interactions are shown schematically in Figure 6. The side chains accommodate the barrier to inter-unit rotation in the polymer backbone by aligning in different ways to maintain contact with the solvent environment. These associations are also displayed in the simulations at 20 °C and 90 °C where phase formation is somewhat more diffuse than those seen at other temperatures. In these simulations, the side chains of the PBS-PF2T units appear to interact with the solvent present within the reverse micellar section. Figure 7 shows a singular scene from the simulation carried out at 20 °C which shows that the surfactant molecules (shown in green) interact predominantly with the backbone whereas the terminal sulfonate groups remain in closer proximity with the surrounding solvent and the side chains show a high degree of distortion to accommodate this sulfonate group-solvent interaction. In order to examine this observation, further analysis was carried out on each of the trajectories generated throughout the study. This was performed by examining the number of contacts below 0.6 nm between the side chains and the surrounding solvent and comparing these to the number of contacts between the side chains and the surfactant. It can be seen that in each case there is a significantly higher number of contacts between the CPE side chains and the solvent than that which exists between the side chains and the surfactant (Tables S5–S8). This strongly supports the observation that the side chains do possess a high degree of hydrophilicity and explains the propensity for the side chains to remain in close contact with the solvent. It is also apparent that the number of contacts between the backbone of PBS-PF2T and the surfactant is also higher than those between the side chains and surfactant which is agreement with the perceived orientation of the PBS-PF2T units in relation to the surfactant.

**Figure 8:**
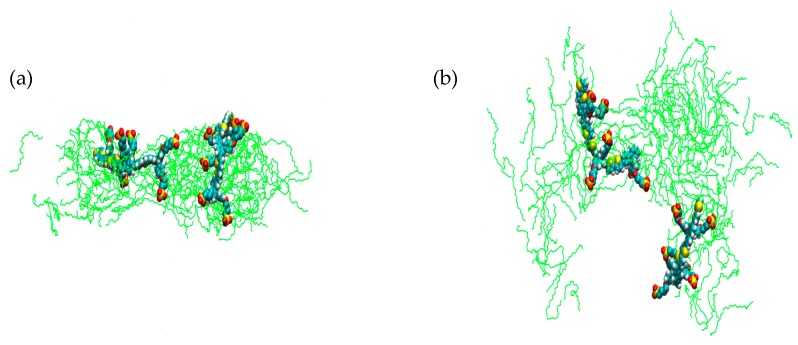
Simulated annealing cell representation of (**a**) front view; and (**b**) side view of 680 mM C_12_E_5_ with two equivalents of PBS-PF2T at 20 °C after 10 ns (PBS-PF2T is shown in van der Waals representations and solvent is omitted for clarity).

**Figure 9:**
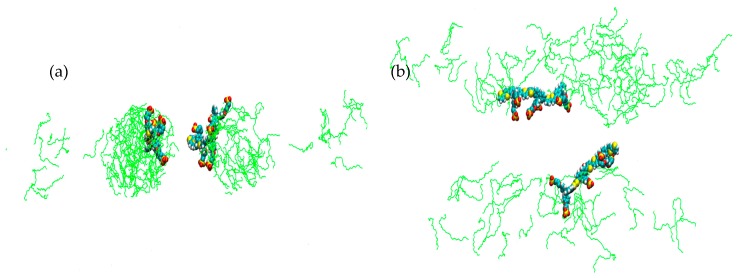
Simulated annealing cell representation of (**a**) front view; and (**b**) side view of 680 mM C_12_E_5_ with two equivalents of PBS-PF2T at 10 °C after 35 ns (PBS-PF2T is shown in van der Waals representations and solvent is omitted for clarity).

In order to test the validity of using MDS as a method by which to examine phase formation, a number of simulations in addition to those shown here were performed. These additional simulations were started from different starting arrangements in the simulations and were also performed at longer simulation times of 20 ns in order to determine the persistence of the observed phases (Tables S2–S4). A complete set of runs at all temperatures from a different arrangement of starting molecules (Figure S16), structure B systems **1b**–**6b**, was performed and in each case the same range of phases were observed for each temperature showing a very promising agreement (Figures S4a,b, S5a,b, S6a,b, S7a,b, S8a,b and S9a,b). Simulations were then performed at 0 °C and 70 °C from a third starting arrangement, structure C, (Figures S4e,f and S8c,d). At 0 °C the same cylindrical type assembly is seen, but for 70 °C an isotropic phase is seen. When a simulation starting from a fourth different starting arrangement, structure D, is performed at 70 °C the cylindrical arrangement is regenerated. A possible explanation for this could be that when the phase diagram for phases in the binary water-C_12_E_5_ system is considered there is a phase boundary which exists between the isotropic L_1_ and L_3_ phases and L_α_ lamellar phase at 25% C_12_E_5_ concentration at ~70 °C (Figure S17) [35]. Although the phase type created in the simulations differs in type to that reported in the phase diagram for C_12_E_5_, the temperatures of the phase boundaries show a good agreement. The close proximity of differing phases at ~70 °C could provide a description of the inconsistency observed in the MDS at the same temperature as it is possible that at this temperature a phase boundary exists which could account for the observation of both cylindrical and isotropic phased shown in the simulations. A simulation was also performed at 45 °C from structure D and again consistency was observed by the presence of the cylindrical assembly seen in the other simulations at that temperature (Figure S7c,d). The findings from these simulations agree strongly with experimental results from the previous study of PBS-PF2T in C_12_E_4_ [27]. Additionally, a simulated annealing was performed between 20 °C and 10 °C (details of simulation in Supplementary Materials) to show how the phase changes between each of these temperatures, Figure 8 shows the system at 20 °C which is isotropic in appearance whereas Figure 9 shows the system at 10 °C showing the formation of two cylindrical PBS-PF2T:C_12_E_5_ assemblies, this is consistent with what was observed for individual simulations at these temperatures and provides further validation for the use of MDS as a method to investigate temperature effects on phase formation.

Fluorescence measurements on PBS-PF2T in C_12_E_4_ showed a blue shifted emission maximum which is indicative of inhibited aggregation of PBS-PF2T. The accompanying SAXS measurements and visual observations also suggested that, in the case of PBS-PF2T: A C_12_E_4_/water mixture, at temperatures below 20 °C, micelle formation is observed which leads to a cylindrical phase upon heating to 70 °C. This phase persists at higher temperatures with an apparent dissociation of PBS-PF2T. It is justifiable here to state that it is indeed possible to influence phase formation in aqueous CPE:surfactant systems by alteration of temperature and also using different surfactant types. However, it is not possible to state conclusively, using MDS, that addition of surfactant interrupts π-π interactions between adjacent PBS-PF2T as the united atom force fields employed here do not have a definitive explanation of the electrons or polarization which are involved in such attractive π-π effects.

## 3. Discussion

This study has provided clear evidence for the breakup of PBS-PF2T aggregation upon addition of the nonionic surfactant C_12_E_5_, and can be extended to the behavior with other related alkyloxyethylene surfactants. Figure 3 shows a previously studied simulation of pure PBS-PF2T in a water environment and clearly demonstrates aggregate formation occurring between the 10 PBS-PF2T equivalents. When this simulation of pure PBS-PF2T is compared to those containing PBS-PF2T in aqueous C_12_E_5_/water environments we can see that this aggregation is inhibited and this is followed by a characteristic phase formation. The formation of rod-like cylinder phases dominates at 0 °C, 10 °C, 45 °C and 70 °C. At 20 °C and 90 °C there is a less defined phase formation, which has some semblance of a reverse micellar phase where there is a circular-type hydrophilic region within which there appears to be an isotropic layer. These findings are in very strong agreement with those which were found in simulations where C_12_E_4_ was the surfactant. In the case of C_12_E_4_, at 10 °C a micellar phase was seen and at 20 °C, 45 °C, 70 °C and 90 °C phase formation occurred in the nature of rod-like lamellar structures. In all simulations, whether the surfactant present is C_12_E_5_ or C_12_E_4_, it is important to observe that the PBS-PF2T species do not interact with each other and resultantly no CPE:CPE (PBS-PF2T:PBS-PF2T) aggregates are formed but instead form distinct polymer:surfactant assemblies. Another very important observation relates to the orientation of the PBS-PF2T units which is shows a marked level of specificity and demonstrates an affinity for the backbone of the polyelectrolyte to interact with the surfactant assembly and the side chains to remain exposed to the surrounding solvent environment. These results suggest that it is possible to extend the MD simulations to systems involving alkyloxyethylene surfactants of other alkyl or ethylene oxide chain lengths. 

## 4. Materials and Methods

### 4.1. Molecular Dynamics Simulations

Molecular dynamics simulations were performed using the GROMACS version 4.5.5 software (Department of Biophysical Chemistry, University of Groningen, Groningen, The Netherlands) package with the standard GROMOS96 53a6 force field [37,38,39,40]. Simulations were performed on x86_64, 8 CPU, Intel Core i7-3770 3.40 GHz processor system.

Structures for PBS-PF2T and C_12_E_5_ were built and minimized using the Avogadro molecular builder (Univeristy of Pittsburgh, Pittsburgh, PA, USA) [40], protein data bank files of minimized structures were then submitted to the automated topology builder [41] to generate the necessary topology files for simulations using GROMACS, the automated topology builder has been successfully applied in other studies on similar systems involving conjugated polyelectrolytes [26,27].

Two equivalents of PBS-PF2T were added to a cubic box of 7 × 7 × 7 nm^3^ to which 680 mM C_12_E_5_ was added. The remainder of the cell was taken up by water by employing the SPC solvation model [42] which considers a simple three-point charge model for water where the intramolecular degrees of freedom are frozen and the intermolecular interactions are described by a combination of Lennard-Jones and Coulombic potentials between sites of fixed point charges. The box was then constrained in accordance with the LINCS algorithm [43]. Simulations were carried out over a time frame of 10 ns with a step size of 2 fs. All visualizations and images were generated using VMD software [44]. Output of the simulations were converted using the trjconv command with –pbc nojump and –center options specified in GROMACS.

The SPC model used represents, with accuracy, the properties of a bulk water environment under standard conditions, 300 K and 1 atmosphere pressure, and was selected although these systems involve the investigation of temperature increase. The SPC model has also been employed in the study of similar CPEs in aqueous environments.

### 4.2. Computational Details

The number of C_12_E_5_ molecules required to fill 25% (680 mM) of the cell of dimensions 7 × 7 × 7 nm^3^ was determined by calculating the weight of the cell containing the two equivalents of PBS-PF2T and the rest of the volume of the cell is filled with water (2 equivalents of PBS-PF2T and 11,010 H_2_O molecules had a total weight 203,112 g·mol^−1^) it could then be calculated that, in order for 25% (680 mM)of this volume to contain non-ionic surfactant, 126 equivalents of C_12_E_5_ needed to be added (203,112 × 0.25 = 50,778 g·mol^−1^ → 50,778/403 g·mol^−1^ = 126 equivalents of C_12_E_5_).

In the case of simulations with C_12_E_4_ present as the surfactant, cells were 10 × 10 × 10 nm^3^ in dimensions and The number of C_12_E_4_ molecules required to fill 680 mM of the cell of dimension 10 × 10 × 10 nm^3^ was determined by calculating the weight of the cell containing the two equivalents of PBS-PF2T and the rest of the volume of the cell is filled with water (two equivalents of PBS-PF2T and 29,744 H_2_O molecules had a total weight 540,104 g·mol^−1^) it could then be calculated that, in order for 680 mM of this volume to contain non-ionic surfactant, 373 equivalents of C_12_E_4_ needed to be added (540,104 × 0.25 = 135,026 g·mol^−1^ → 135,026/362 g·mol^−1^ = 373 equivalents of C_12_E_4_). The simulation of PBS-PF2T was also performed in a cell of 10 × 10 × 10 nm^3^ containing 10 equivalents of PBS-PF2T and 22,992 H_2_O molecules. In order to obtain overall system neutrality 12 Na^+^ ions were added in each simulation.

### 4.3. Molecular Dynamics Details

Below are the criteria specified within the dynamics simulations. Equilibration was determined using g_energy command and all systems were found to equilibrate rapidly. All simulations, unless otherwise specified were run over 10 ns with a timestep of 2 fs. In the trajectory run all simulations were performed with periodic boundary conditions, using the Berendsen coupling algorithm [45]. The particle mesh Ewald [46] method was used for consideration of long range electrostatic interactions. The following values were also included: nstlist = 10, vdwtype = cut-off, rvdw = 1.4, rcoulomb = 1.0. Prior to each simulation an energy minimization was performed followed by a dynamics equilibration before a full trajectory run. Graph S1 show equilibration of the potential energy, overall energy and temperature of simulation **2b** as an example dynamics run, similar plateauing is observed for the same criteria for each of the other simulations.

## 5. Conclusions

We have been able to show here through the use of molecular dynamics simulations that a solution of conjugated polyelectrolyte PBS-PF2T can be successfully broken up and aggregation impeded in the presence of 680 mM C_12_E_5_ solution. Not only is cluster formation prevented but it is also seen that the temperature and the nature of the surrounding surfactant can influence the type of phase formed. In simulations of PBS-PF2T in C_12_E_4_ and C_12_E_5_, micellar, cylindrical and more diffuse phases occur depending upon the temperature of the system and the surfactant; further investigation will consist of examination of higher concentrations of both CPE and surfactant in order to determine whether at such higher concentrations are multiple cylindrical/micellar phases formed and in so doing can we redefine the phase formed and consider a true liquid crystalline phase to be formed. The presence of what at this point appears to be a reverse micellar phase seen at 20 °C and 90 °C in the case of a C_12_E_5_ environment is an extremely interesting observation in terms of nano- and self-assembly as such vesicles could be utilized as a means of protein or ion transport in biological systems and could open a wider range of applications for these CPE:Surfactant systems. The simulations performed herein have given a beneficial insight into the behavior of these systems and provide a possible method of investigation by which the disadvantages associated with the use of conjugated polyelectrolytes, such as solubility and reduction in fluorescence yields and red-shifted fluorescence maxima, could be overcome. These findings can be of aid in the understanding of the behaviors of CPEs in different aqueous environments and can be of great use in the design and applicability of such systems in solution based, biological and chemical technologies.

## Supplementary Materials

The following are available online at www.mdpi.com/1996-1944/9/5/379/s1.

## Abbreviations

The following abbreviations are used in this manuscript:

MDS	Molecular Dynamics Simulations
PBS-PF2T	poly[9,9-bis(4-sulfonylbutoxyphenylphenyl) fluorene-2,7-diyl-2,2’-bithiophene]
C12E5	pentaethylene glycol monododecyl ether
C12E4	tetraethylene glycol monododecyl ether (C12E4)
GISAXS	grazing incidence small angle X-ray scattering
SAXS	small angle X-ray scattering
