# Supplementary material for: Molecular Dynamics Study of Self-Assembly of Aqueous Solutions of Poly[9,9-bis(4-Sulfonylbutoxyphenylphenyl) Fluorene-2,7-diyl-2,2’-Bithiophene] (PBS-PF2T) in the Presence of Pentaethylene Glycol Monododecyl Ether (C12E5)
Source: Materials (Basel). 2016 May 18;9(5):379. doi: 10.3390/ma9050379 (PMC5503020; doi:10.3390/ma9050379)
Supplement: Supplementary file 1 [file materials-09-00379-s001.pdf]

Supplementary Materials: Molecular Dynamics Study of Self-Assembly of Aqueous Solutions of Poly[9,9-bis(4-Sulfonylbutoxyphenylphenyl) Fluorene-2,7-diyl-2,2′-Bithiophene] (PBS-PF2T) in the Presence of Pentaethylene Glycol Monododecyl Ether ($C_{12}E_5$)

Beverly Stewart and Hugh Douglas Burrows

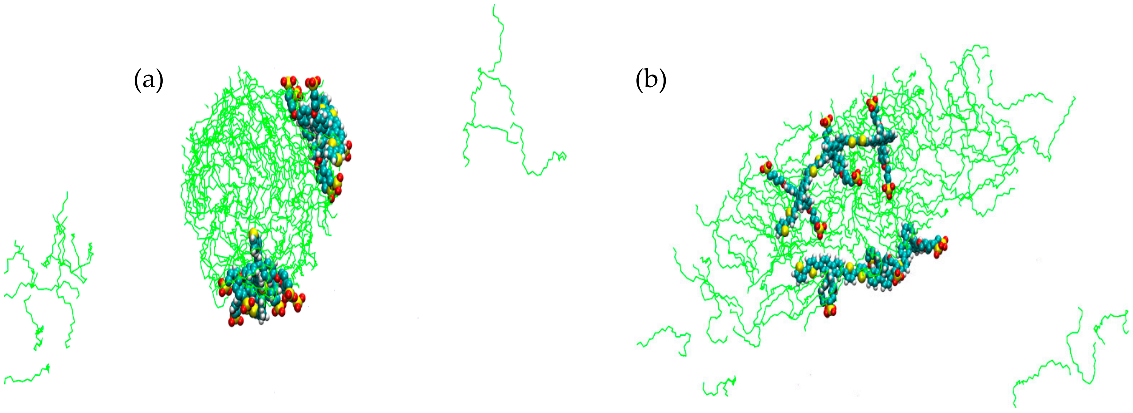

Figure S1. Simulation cell representation (**a**) front view; and (**b**) side view of 680 mM $C_{12}E_5$ with two equivalents of PBS-PF2T at 10 °C after 10 ns, system **2**. (PBS-PF2T is shown in van der Waals representations and solvent is omitted for clarity).

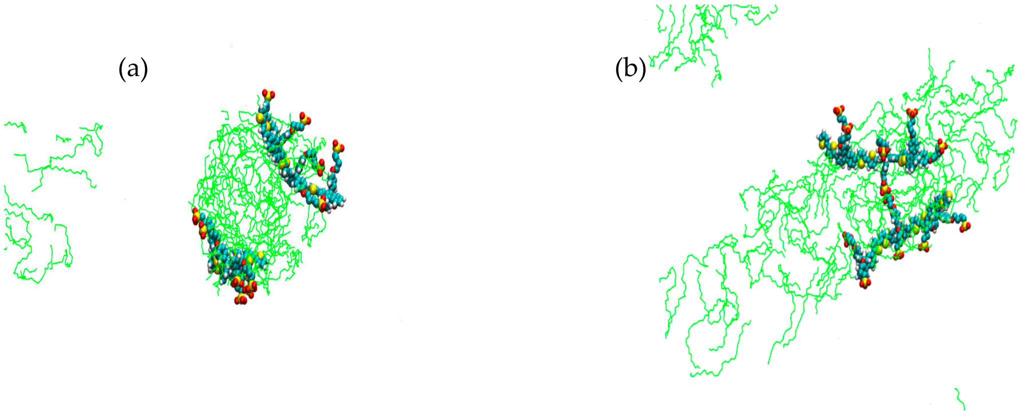

Figure S2. Simulation cell representation of (**a**) front view; and (**b**) side view of 680 mM $C_{12}E_5$ with two equivalents of PBS-PF2T at 45 °C after 10 ns, system **4**. (PBS-PF2T is shown in van der Waals representations and solvent is omitted for clarity).

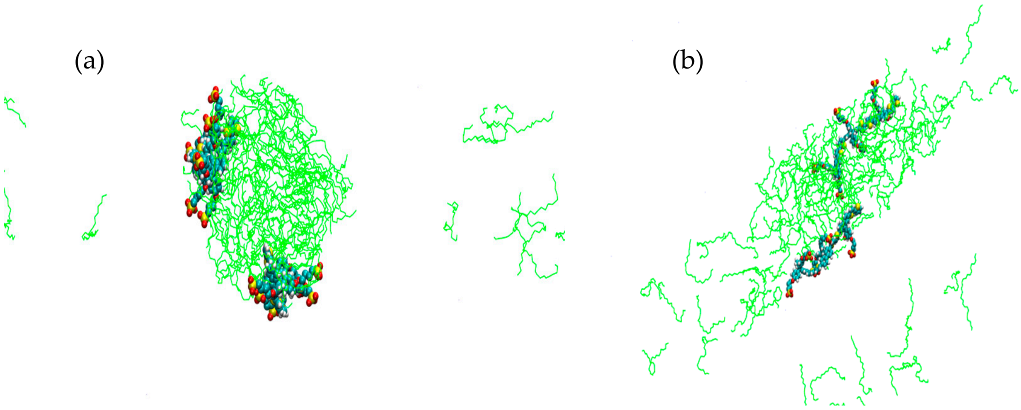

Figure S3. Simulation cell representation of (**a**) front view; and (**b**) side view of 680 mM $C_{12}E_5$ with two equivalents of PBS-PF2T at 70 °C after 10 ns, system **5**. (PBS-PF2T is shown in van der Waals representations and solvent is omitted for clarity).

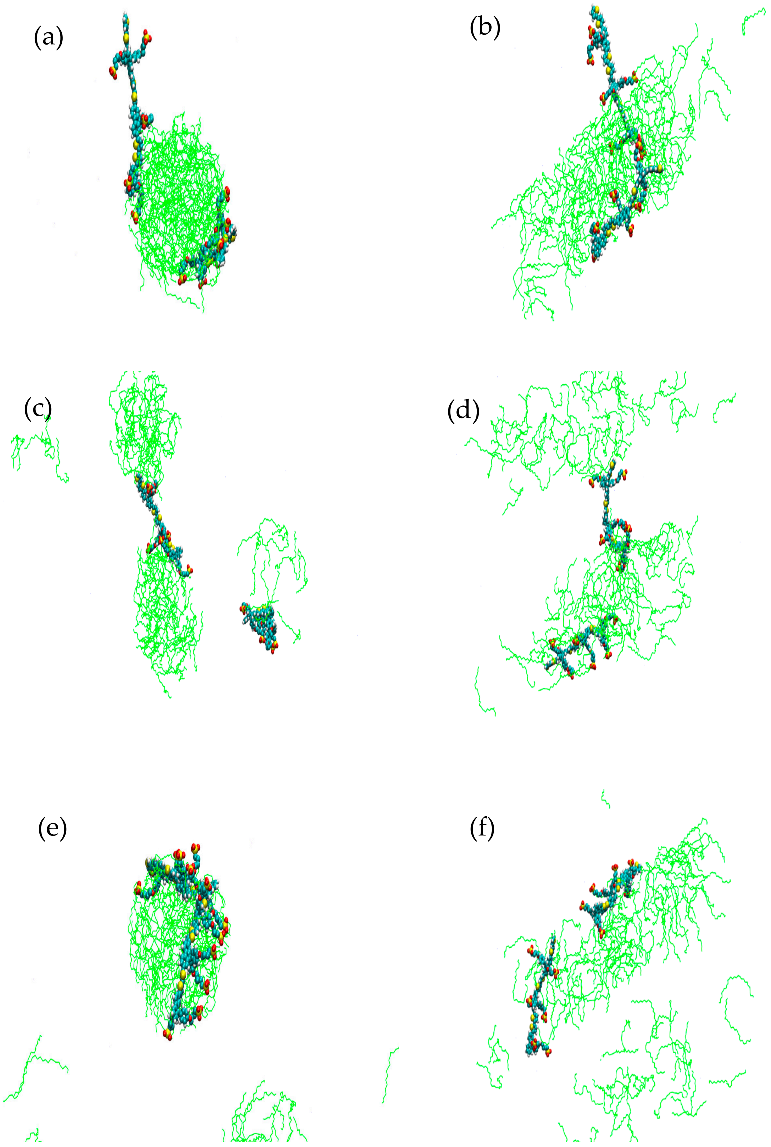

Figure S4. Simulation cell representation of (**a**) front view; (**b**) side view of simulation at 0 °C extended to 20 ns, system **1**. Representation of (**c**) front view; (**d**) side view of simulation at 0 °C from starting structure B at 20 ns, system **1b**. Representation of (**e**) front view; (**f**) side view of simulation at 0 °C from starting structure C at 20 ns, system **1c**.

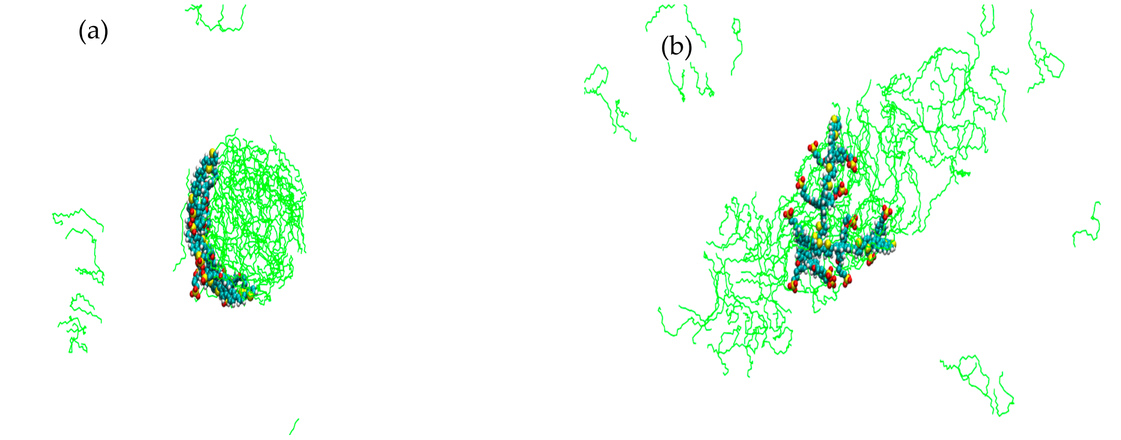

Figure S5. Simulation cell representation of (**a**) front view; (**b**) side view of simulation at 10 °C from starting structure B at 20 ns, system **2b**.

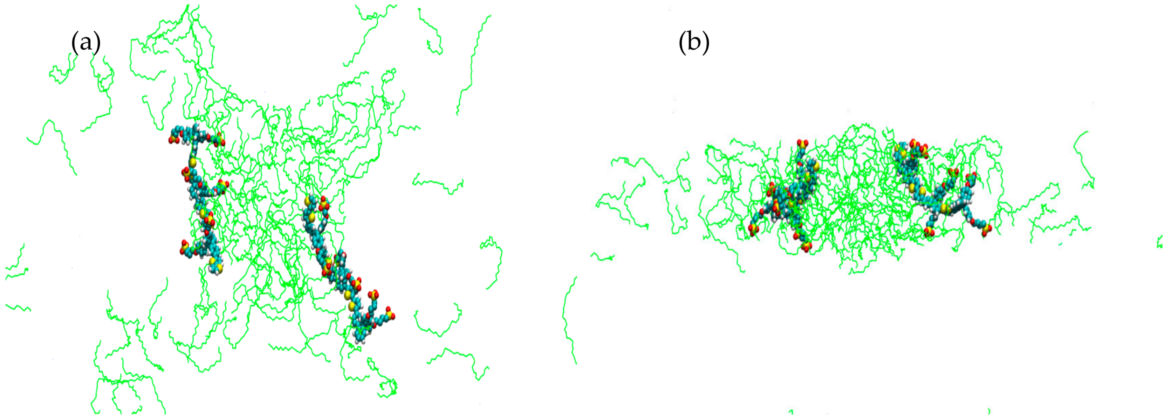

Figure S6. Simulation cell representation of (**a**) front view; (**b**) side view of simulation at 20 °C from starting structure B at 20 ns, system **3b**.

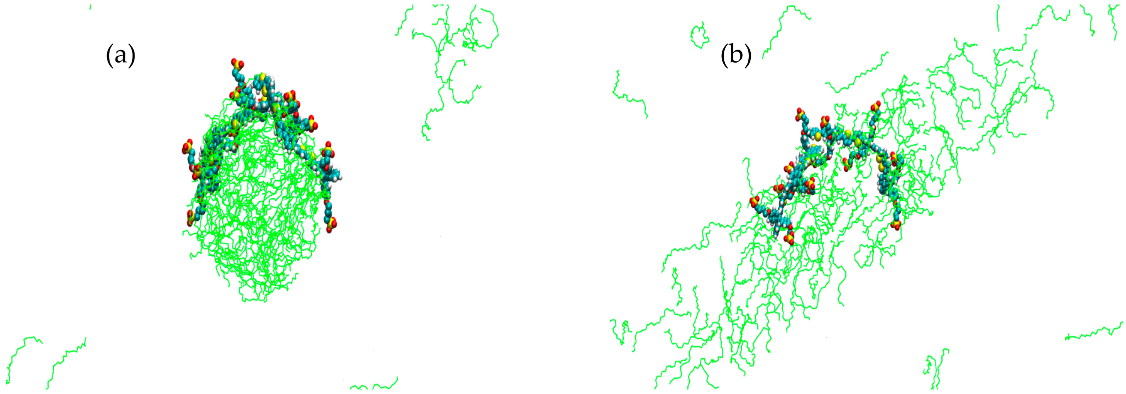

Figure S7. *Cont.*

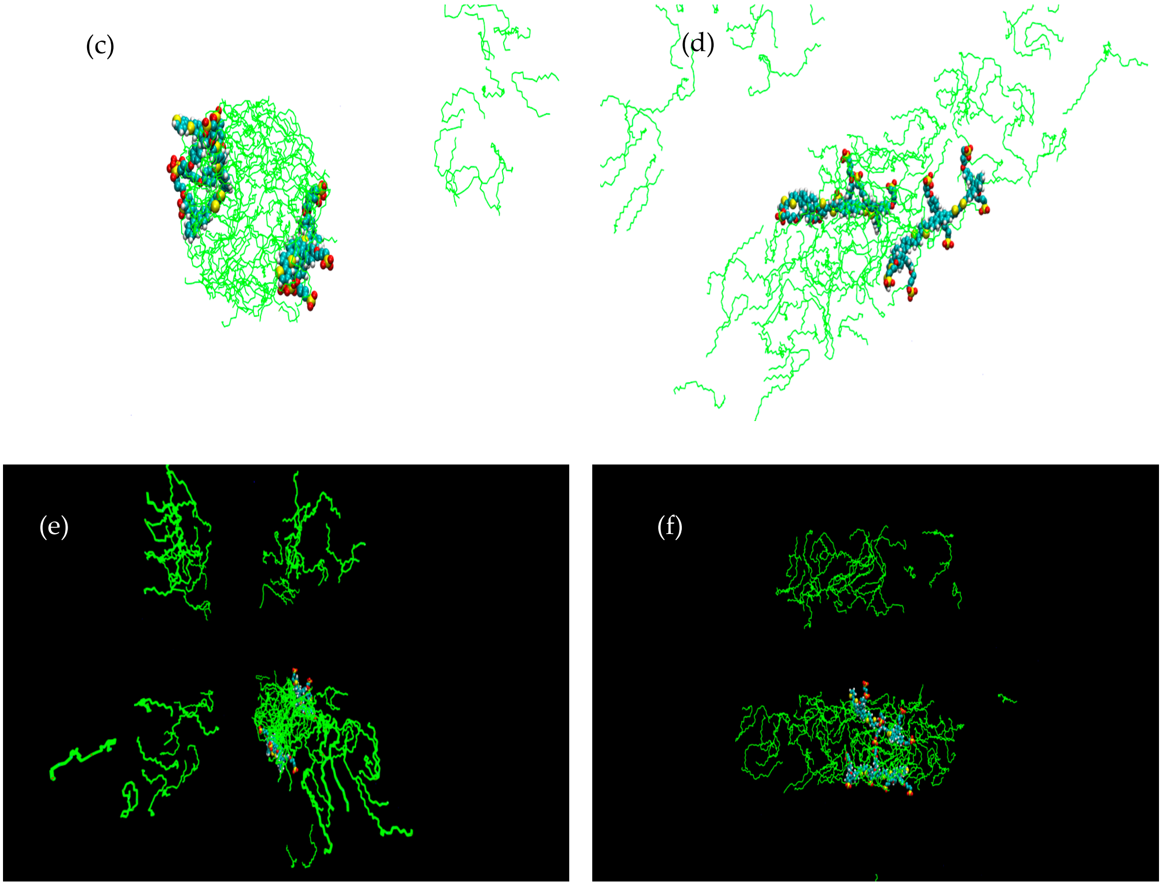

Figure S7. Simulation cell representation of (**a**) front view; (**b**) side view of simulation at 45 °C from starting structure B at 20 ns, system **4b**. Representation of (**c**) front view; (**d**) side view of simulation at 45 °C from starting structure D at 20 ns, system **4d**. Representation of (**e**) front view; (**f**) side view of simulation at 45 °C from original structure at 10 ns with black background showing the formation of three other loosely populated cylindrical arrangements, system **4**.

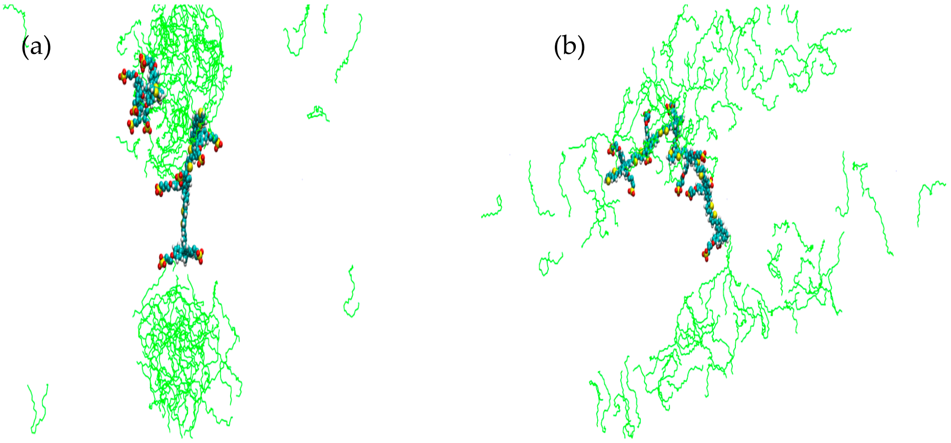

Figure S8. *Cont.*

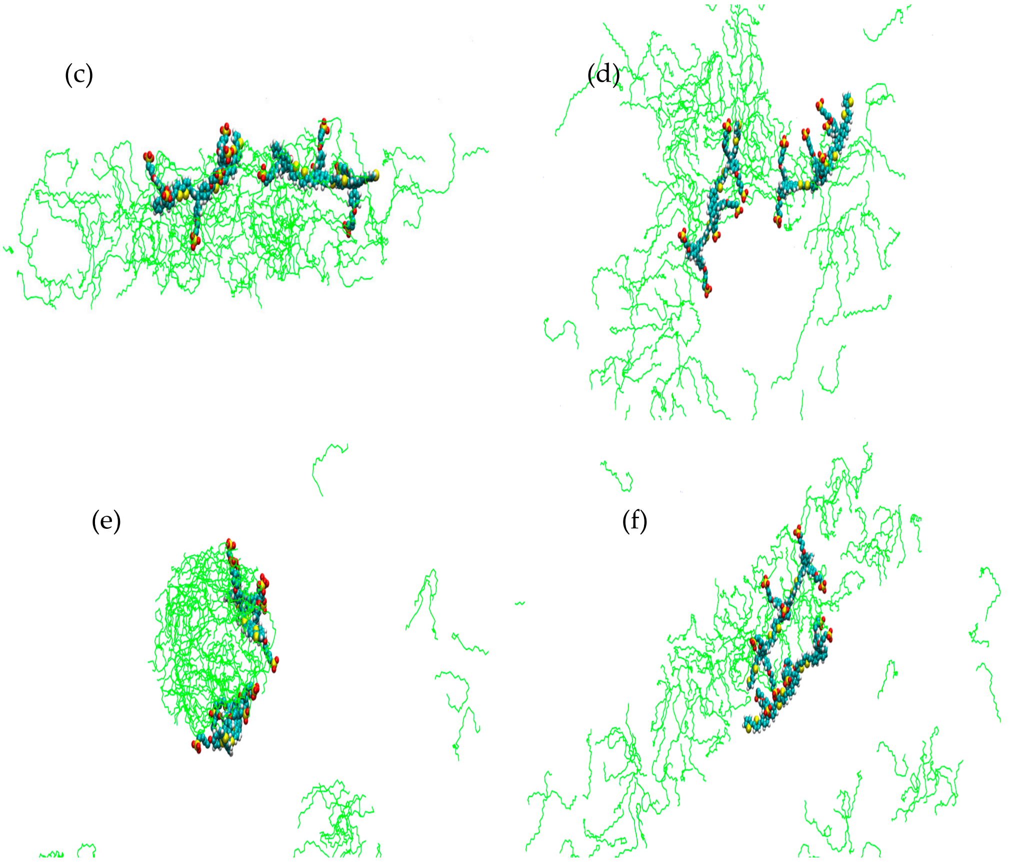

Figure S8. Simulation cell representation of (**a**) front view; (**b**) side view of simulation at 45 °C from starting structure B at 20 ns, system **5b**. Representation of (**c**) front view; (**d**) side view of simulation at 45 °C from starting structure C at 20 ns, system **5c**. Representation of (**e**) front view; (**f**) side view of simulation at 45 °C from starting structure D at 20 ns, system **5d**.

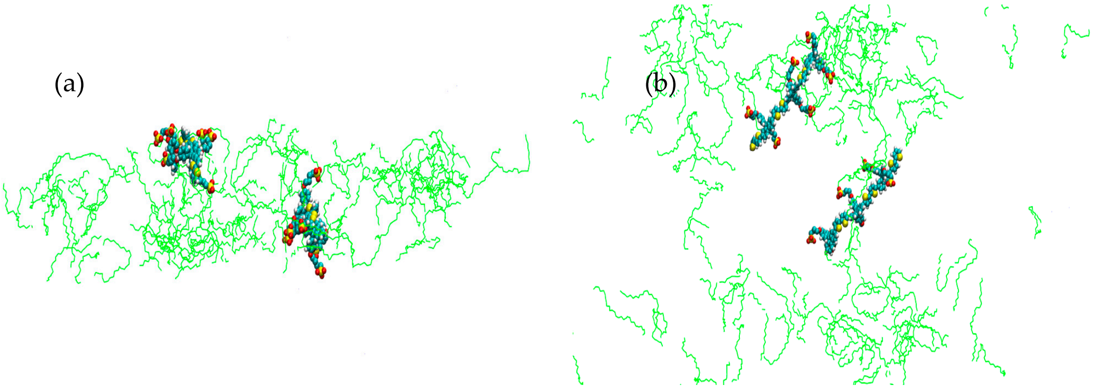

Figure S9. Simulation cell representation of (**a**) front view; (**b**) side view of simulation at 90 °C from starting structure B at 20 ns, system **6b**.

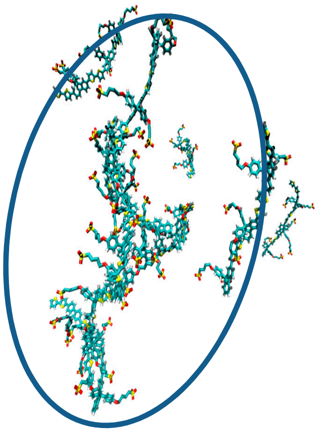

Figure S10. Simulation cell representation of ten equivalents of PBS-PF2T (bonds representation) in water showing formation of chain like elongated aggregate (encircled).

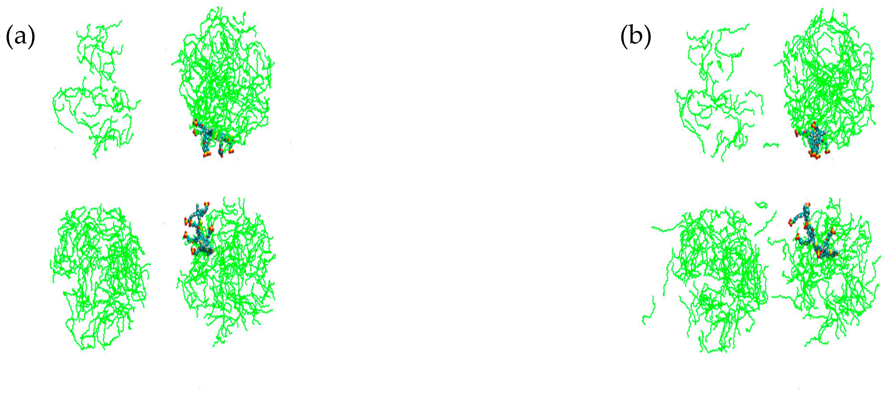

Figure S11. Simulation cell representation of (**a**) front view; (**b**) side view of simulation at 10 °C from starting structure B at 20 ns.

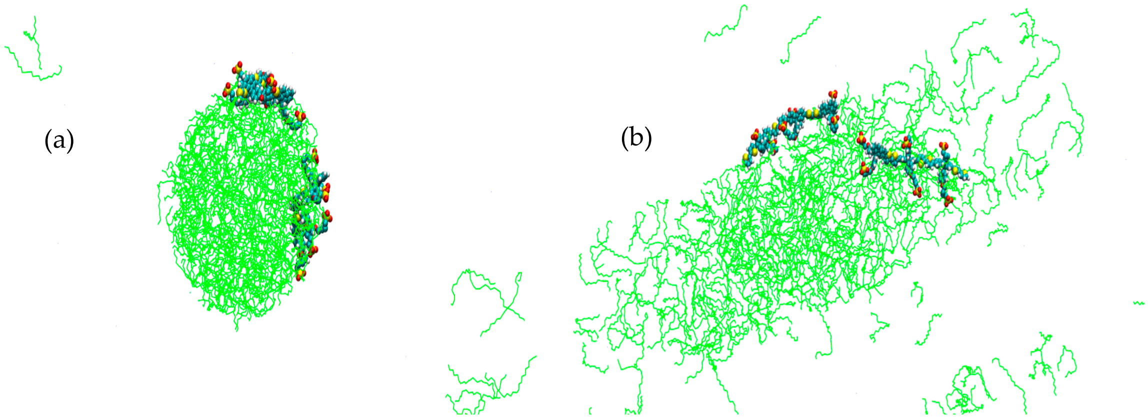

Figure S12. Simulation cell representation of (**a**) front view; (**b**) side view of simulation at 20 °C from starting structure B at 20 ns.

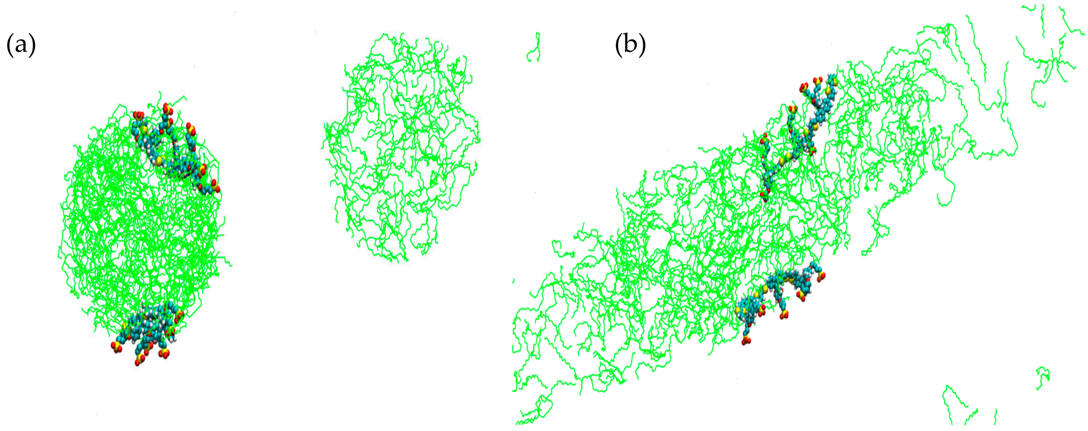

Figure S13. Simulation cell representation of (**a**) front view; (**b**) side view of simulation at 45 °C from starting structure B at 20 ns.

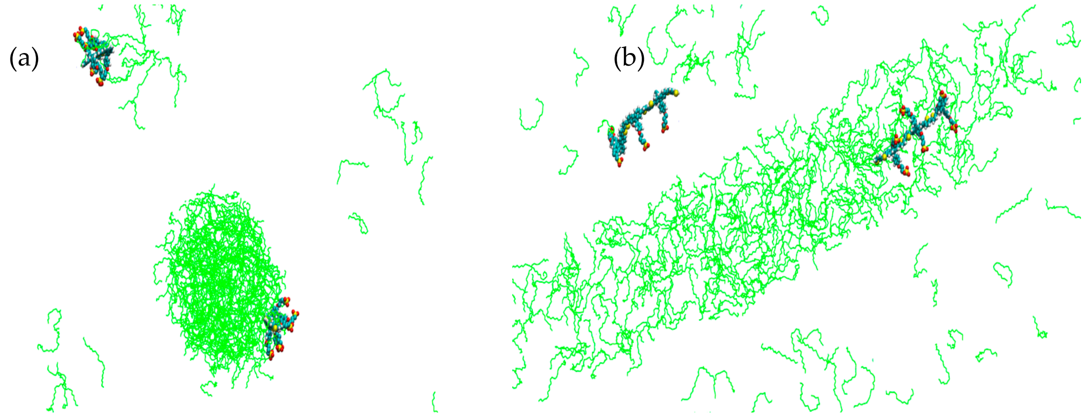

Figure S14. Simulation cell representation of (**a**) front view; (**b**) side view of simulation at 70 °C from starting structure B at 20 ns.

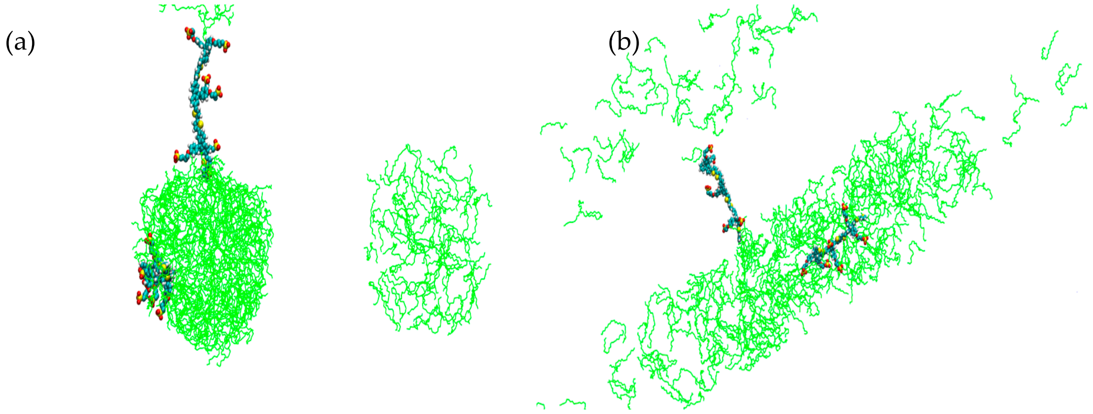

Figure S15. Simulation cell representation of (**a**) front view; (**b**) side view of simulation at 90 °C from starting structure B at 20 ns.

Table S1. System details for the molecular dynamics simulations indicating the composition of the solvent cell in each simulation. In all simulations, unless otherwise specified, the cell was of dimensions 7 × 7 × 7 nm^3.

	**1**	**2**	**3**	**4**	**5**	**6**
PBS-PF2T	2	2	2	2	2	2
Water	7944	7944	7944	7944	7944	7944
Na^+	12	12	12	12	12	12
$C_{12}E_5$	126	126	126	126	126	126
Temperature	0 °C	10 °C	20 °C	45 °C	70 °C	90 °C
Time	10 ns *	10 ns	10 ns	10 ns	10 ns	10 ns

*: this simulation was extended to 20 ns.

Table S2. System details for the molecular dynamics simulations from starting structure B, indicating the composition of the solvent cell in each simulation. In all simulations, unless otherwise specified, the cell was of dimensions 7 × 7 × 7 nm^3.

	**1b**	**2b**	**3b**	**4b**	**5b**	**6b**
PBS-PF2T	2	2	2	2	2	2
Water *	7944	7944	7944	7944	7944	7944
Na^+	12	12	12	12	12	12
$C_{12}E_5$	126	126	126	126	126	126
Temperature	0 °C	10 °C	20 °C	45 °C	70 °C	90 °C
Time	20 ns	20 ns	20 ns	20 ns	20 ns	20 ns

*: Solvent molecules added using –max sol option in gromacs.

Table S3. System details for the molecular dynamics simulations from starting structure C, indicating the composition of the solvent cell in each simulation. In all simulations, unless otherwise specified, the cell was of dimensions 7 × 7 × 7 nm^3.

	**1c**	**5c**
PBS-PF2T	2	2
Water *	7944	7944
Na^+	12	12
$C_{12}E_5$	126	126
Temperature	0 °C	70 °C
Time	20 ns	20 ns

*: Solvent molecules added using –max sol option in gromacs.

Table S4. System details for the molecular dynamics simulations from starting structure D, indicating the composition of the solvent cell in each simulation. In all simulations, unless otherwise specified, the cell was of dimensions 7 × 7 × 7 nm^3.

	**4d**	**5d**
PBS-PF2T	2	2
Water *	7944	7944
Na^+	12	12
$C_{12}E_5$	126	126
Temperature	45 °C	70 °C
Time	20 ns	20 ns

*: Solvent molecules added using –max sol option in gromacs.

Random starting structures were generated by using –random seed option in genbox command.

For the previous stuy where $C_{12}E_4$ was used as the surfactant [27], the number of $C_{12}E_4$ molecules required to fill 25% 680 mM of the cell of dimension 10 × 10 × 10 nm^3 was determined by calculating the weight of the cell containing the two equivalents of PBS-PF2T and the rest of the volume of the cell is filled with water (2 equivalents of PBS-PF2T and 29,744 H_2O molecules had a total weight 540,104 g·mol^{-1}) it could then be calculated that, in order for 25% 680 mM of this volume

to contain non-ionic surfactant, 373 equivalents of $C_{12}E_4$ needed to be added (540,104 × 0.25 = 135,026 g·mol^{-1} → 135,026/362 g·mol^{-1} = 373 equivalents of $C_{12}E_4$). For the simulation of ten equivalents of PBS-PF2T, ten PBS-PF2T species were added to a simulation cell of 10 × 10 × 10 nm^3 with 60 Na^+ ions and 22,992 solvent molecules.

Simulated annealing was performed between 10 °C and 20 °C, the entire system was coupled to 293 K (20 °C) from system 3 and a trajectory was run at 293 K (20 °C) for 10 ns, between 10 and 15 ns the system was cooled to 283 K (10 °C) and the trajectory was run for a further 20 ns making a total simulation time of 35 ns.

Table S5. Showing the calculated number of contacts below 0.6 nm in the first and final frames of the simulation demonstrating the closer proximity of the side chains with the solvent for systems **1**–**6**. Contacts were calculated using the g_mindist command in gromacs.

	0 °C (1)		10 °C (2)		20 °C (3)		45 °C (4)		70 °C (5)		90 °C (6)	
	Initial	Final	Initial	Final	Initial	Final	Initial	Final	Initial	Final	Initial	Final
Side Chain–Solvent	5938	4300	5938	3872	5938	3763	5938	3962	5938	4199	5938	4396
Side chains–Surfactant	392	1335	392	1555	392	1594	392	1490	392	1250	392	1056
Backbone–Solvent	8967	6840	8967	4434	8967	2682	8967	2590	8967	3049	8967	1638
Backbone–Surfactant	447	1930	447	3103	447	3779	447	3651	447	3086	447	3701

Table S6. Showing the calculated number of contacts below 0.6 nm in the first and final frames of the simulation demonstrating the closer proximity of the side chains with the solvent for systems **1b**–**6b**. Contacts were calculated using the g_mindist command in gromacs.

	0 °C (1b)		10 °C (2b)		20 °C (3b)		45 °C (4b)		70 °C (5b)		90 °C (6b)	
	Initial	Final	Initial	Final	Initial	Final	Initial	Final	Initial	Final	Initial	Final
Side Chain–Solvent	5997	4485	5997	3648	5997	3919	5997	3889	5997	4508	5997	3969
Side chains–Surfactant	398	1287	398	1699	398	1488	398	1473	398	1029	398	1128
Backbone–Solvent	8943	4720	8943	5324	8943	2081	8943	4183	8943	2949	8943	3956
Backbone–Surfactant	440	2996	440	2220	440	4084	440	3072	440	3356	440	2528

Table S7. Showing the calculated number of contacts below 0.6 nm in the first and final frames of the simulation demonstrating the closer proximity of the side chains with the solvent for system **1c** and **2c**. Contacts were calculated using the g_mindist command in gromacs.

	0 °C (1c)		70 °C (2c)	
	Initial	Final	Initial	Final
Side Chain–Solvent	5908	4192	5908	4407
Side chains–Surfactant	511	1466	511	1383
Backbone–Solvent	8920	3025	8920	2419
Backbone–Surfactant	400	3465	400	4044

Table S8. Showing the calculated number of contacts below 0.6 nm in the first and final frames of the simulation demonstrating the closer proximity of the side chains with the solvent for systems **1d** and **2d**. Contacts were calculated using the g_mindist command in gromacs.

	**45 °C (1d)**		**70 °C (2d)**	
	**Initial**	**Final**	**Initial**	**Final**
Side Chain–Solvent	6007	4420	6007	3944
Side chains–Surfactant	426	1116	426	1308
Backbone–Solvent	9001	3894	9001	3449
Backbone–Surfactant	384	2706	384	3207

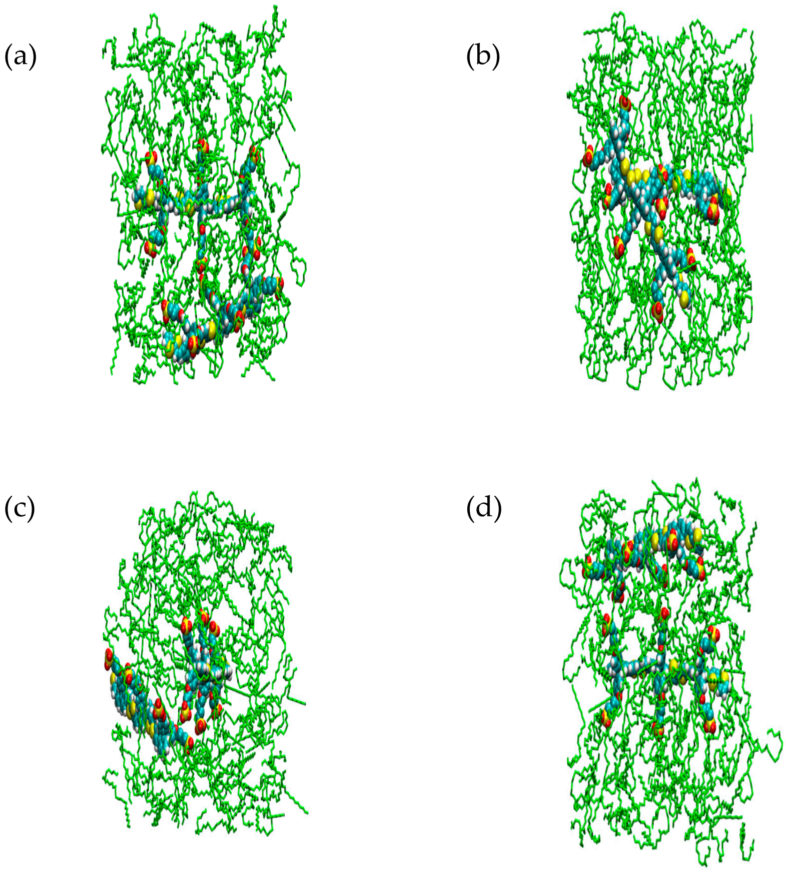

Figure S16. Initial simulation cell representation at t = 0 of (**a**) Structure A; (**b**) Structure B; (**c**) Structure C and (**d**) Structure D.

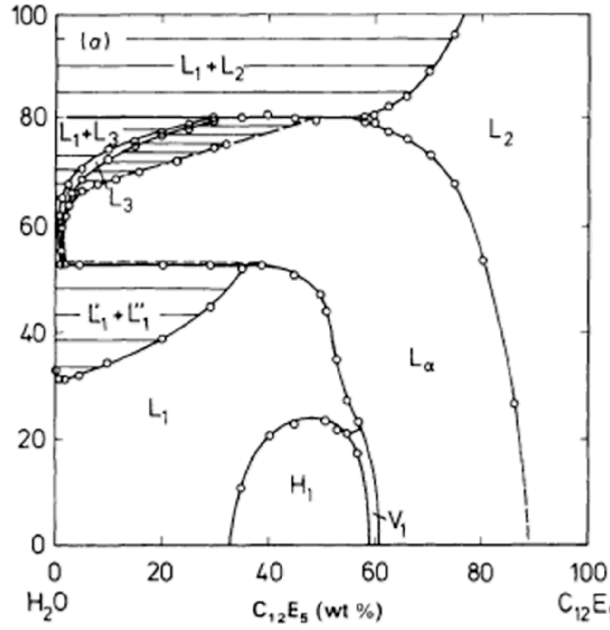

Figure S17. Phase diagram of the water $C_{12}E_5$ system, L_1, L_2 and L_3 denote isotropic iquid solutions, H_1 is a normal hexagonal phase, *V* is a cubic liquid crystalline phase and L_α denotes a lamellar liquid crystalline phase. Full diagram is in the temperature range 0–100 °C. Reproduced from Ref. [35] with permission from the Royal Society of Chemistry.

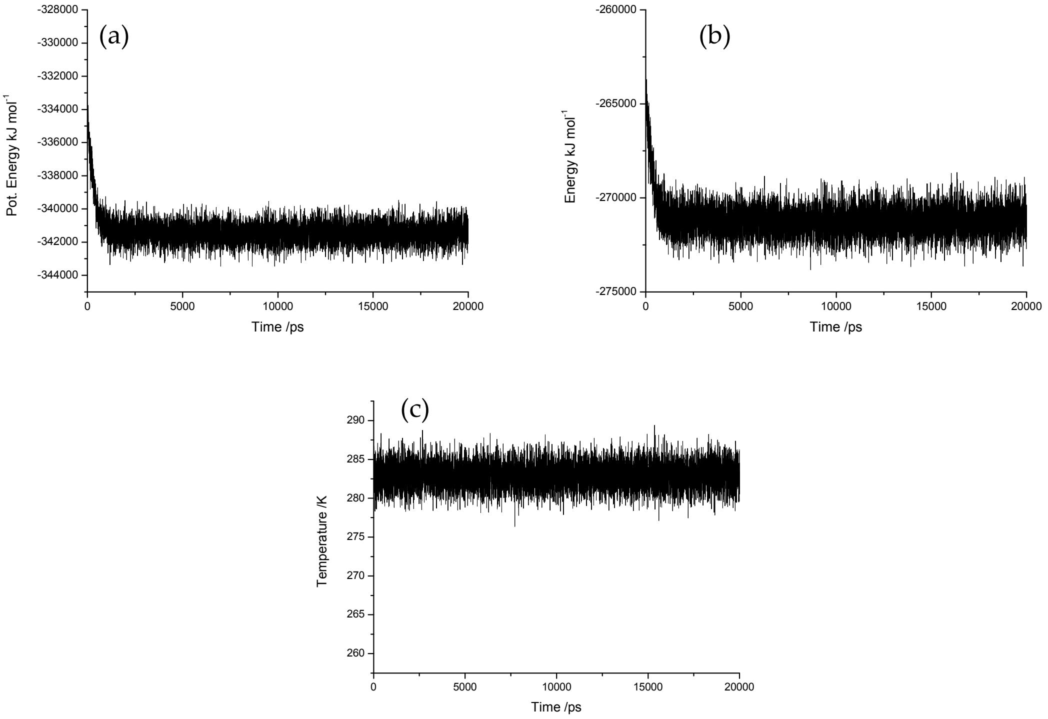

Graph S1. Graph showing (**a**) potential energy; (**b**) energy; and (**c**) temperature of simulation **2b**. In each case equilibration occurs rapidly and remains at a constant energy throughout the simulation and also persists close to the target temperature.

Micropaprticles applied into the central lumen. Scale bar is 1 cm.
